# LMP2-mRNA lipid nanoparticle sensitizes EBV-related tumors to anti-PD-1 therapy by reversing T cell exhaustion

**DOI:** 10.1186/s12951-023-02069-w

**Published:** 2023-09-08

**Authors:** Yu Xiang, Miaomiao Tian, Juan Huang, Yueyi Li, Guangqi Li, Xue Li, Zedong Jiang, Xiangrong Song, Xuelei Ma

**Affiliations:** 1 Department of Biotherapy, Cancer Center, West China Hospital, Sichuan University, Chengdu, Sichuan China; 2 Department of Critical Care Medicine, State Key Laboratory of Biotherapy, West China Hospital, Sichuan University, Chengdu, Sichuan China; 3 Department of Hematology, Sichuan Academy of Medical Sciences and Sichuan Provincial People’s Hospital, University of Electronic Science and Technology of China, Chengdu, Sichuan China; 4 Department of Neurology, West China Hospital, Sichuan University, Chengdu, Sichuan China; 5 Department of Biotherapy, Cancer Center and State Key Laboratory of Biotherapy, West China Hospital, Sichuan University, Chengdu, Sichuan China

**Keywords:** Lymph node targeting, mRNA vaccine, EBV, Nasopharyngeal carcinoma, PD-1, Immunotherapy, Combined therapy

## Abstract

**Background:**

Targeting EBV-proteins with mRNA vaccines is a promising way to treat EBV-related tumors like nasopharyngeal carcinoma (NPC). We assume that it may sensitize tumors to immune checkpoint inhibitors.

**Results:**

We developed an LMP2-mRNA lipid nanoparticle (C2@mLMP2) that can be delivered to tumor-draining lymph nodes. C2@mLMP2 exhibited high transfection efficiency and lysosomal escape ability and induced an increased proportion of CD8 + central memory T cells and CD8 + effective memory T cells in the spleen of the mice model. A strong synergistic anti-tumor effect of C2@mLMP2 in combination with αPD-1 was observed in tumor-bearing mice. The mechanism was identified to be associated with a reverse of CD8 + T cell exhaustion in the tumor microenvironment. The pathological analysis further proved the safety of the vaccine and the combined therapy.

**Conclusions:**

This is the first study proving the synergistic effect of the EBV-mRNA vaccine and PD-1 inhibitors for EBV-related tumors. This study provides theoretical evidence for further clinical trials that may expand the application scenario and efficacy of immunotherapy in NPC.

**Graphical Abstract:**

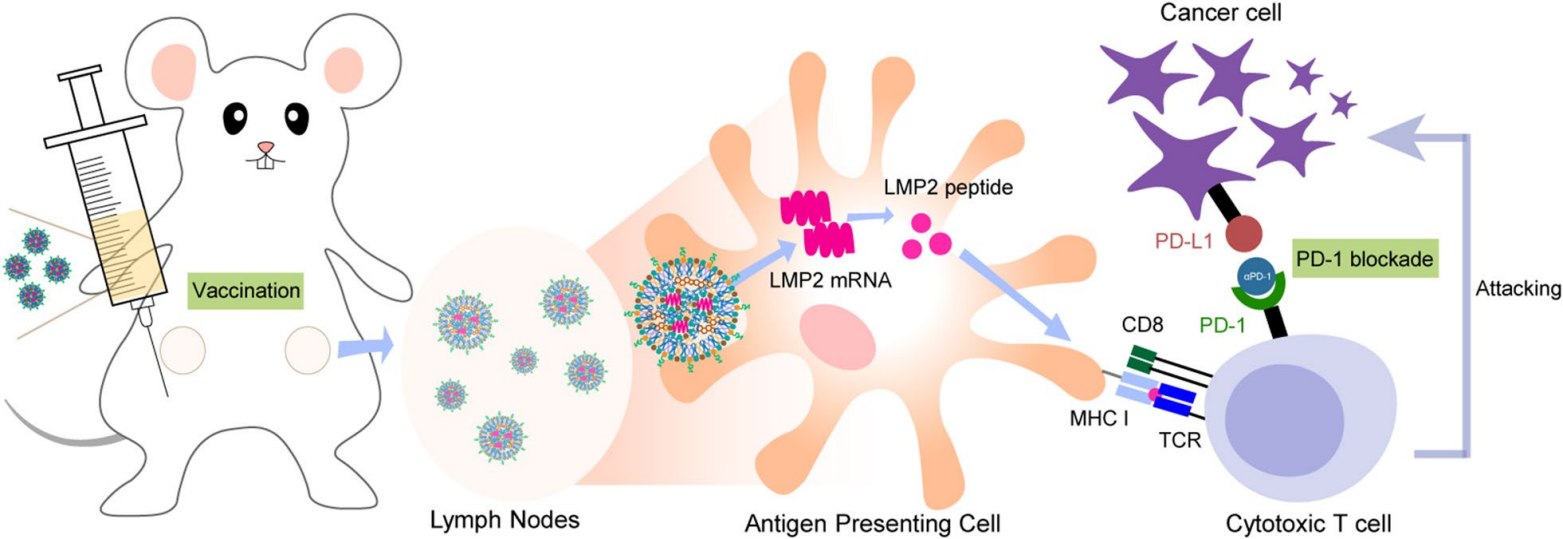

**Supplementary Information:**

The online version contains supplementary material available at 10.1186/s12951-023-02069-w.

## Introduction

Epstein-Barr virus (EBV) is the first human oncogenic virus discovered [1]. More than 90% of the world's population has been infected with EBV [2]. In most cases, the host remains asymptomatic for life [3]. However, there are still some infections that develop into EBV-associated tumors, including nasopharyngeal carcinoma (NPC) [4]. EBV induces a type II latent infection in host cells. The main viral proteins include core antigen EBNA1, latent membrane protein LMP1, and LMP2 [5–7]. These viral proteins induce intracellular signal distortion and promote tumor cell survival, invasion, and metastasis, resulting in a poor prognosis [8]. Considering the important role EBV plays in tumor progression, targeting EBV-proteins becomes a new strategy in NPC treatment [9, 10].

The common treatment methods for NPC include radiotherapy, chemotherapy, surgery or combined therapy [11]. However, due to the invasiveness and asymptomatic nature of NPC, most patients are diagnosed at an advanced stage with local spread [12]. The treatment resistance and toxicity still hinder the therapy of nasopharyngeal cancer [13, 14]. NPC is often accompanied by chronic EBV infection with massive lymphocyte infiltration, high expression of programmed cell death-ligand 1 (PD-L1), and deregulation of T lymphocyte activation [15]. These characteristics determine that NPC is may benefit from immune checkpoint blockade, which blocks immune checkpoint interactions like programmed death receptor-1 (PD-1) and its ligand PD-L1 to cut off immunosuppressive signals from tumor cells and reverse T cell exhaustion [16]. Anti-PD-1 (αPD-1) immunotherapy has been used to treat local recurrence and/or metastatic NPC (R/M-NPC) and achieved great improvement [17, 18]. However, immune checkpoint blockade faces many difficulties such as response heterogeneity, resistance, and intricate immunosuppression pathways. The αPD-1 monotherapy has been recommended as a secondary or late-line choice after platinum-based chemotherapy. Therefore, it is essential to find new combination strategies to enhance the efficacy of αPD-1.

For EBV-driven tumors, vaccines using EBNA1, LMP1, and LMP2 as antigens can induce enhanced anti-tumor immunity [10]. Messenger RNA vaccine has attached attention because of its advantage in tolerability, safety, rapid production, and excellent immune activation ability [19]. We speculated that the combination of EBV-related mRNA vaccine with αPD-1 therapy may induce a potent and long-lasting anti-tumor effect, to the best of our knowledge, very little is known about this. Lymph nodes are the main site where the tumor vaccine works [20] and tumor-draining lymph nodes (TDLN) are also important for αPD-1 response. Therefore, we developed an ionizable lipid nanoparticle (LNP) to deliver the EBV-mRNA LMP2 to the TDLN. Targeted lymph node delivery can reduce side effects and increase the immune response [21]. The LMP2-mRNA is expressed and presented by antigen-presenting cells in the lymph node, which then activates CD8 + T cells to attack cancer cells expressing LMP2. At the same time, αPD-1 is used to cut off the inhibiting signal from tumor PD-L1, achieving a synergistic anti-tumor effect.

## Materials and methods

### Materials and reagents

1,2-Dioctadecanoyl-sn-glycero-3-phophocholine (DSPC,batch number F20130005), Cholesterol (Chol, batch number B90867) and 1,2-dimyristoyl-rac-glycero-3-methoxypolyethylene glycol-2000 (DMG-PEG-2000, batch number 160743-62-4) were purchased from A.V.T. (Shanghai) Pharmaceutical Co.,Ltd,. EGFP mRNA, Lucifrase mRNA, and microfluidic chip were purchased from WestGene Biopharma Co., Ltd. Luciferase substrate was purchased from Yeasen Biotechnology (Shanghai) Co., Ltd. Citric Acid (batch number 100820210302) and Sodium Citrate (batch number 001220210302) were purchased from Hunan Er-Kang Pharmaceutical Co., Ltd. FITC-anti-mouse CD45 and BV711 anti-mouse TIGIT were purchased from Becton Dickinson and Company (New York, USA). APC/Cyanine7-anti-mouse CD3, APC-anti-mouse CD8, PerCP/Cyanine5.5-anti-mouse CD279 (PD-1), PE-anti-mouse CD44, and PE/Cyanine7-anti-mouse CD62L were purchased from Biolegend Inc. (San Diego, CA, USA). Lipofectamine 2000 was purchased from Thermo Fisher Scientific Inc. (MA, USA). DC 2.4 cell was purchased from American Type Culture Collection (ATCC). The Cy5-mRNA was obtained from Vistin (Chengdu, China). The CT26 cell line expressing EBNA1, LMP2, and LMP1 antigens (EBV-CT26) was constructed and provided by Genewiz Inc. Fetal bovine serum (FBS) was obtained from ExCell Biotech (Taicang) Co., Ltd. Penicillin and streptomycin were purchased by Hyclone (Logan, UT, USA). Dulbecco's Modified Eagle Medium (DMEM) was purchased from Cytiva (Marlborough, MA, USA). The anti-mouse PD-1 (αPD-1) drug was provided by Junshi Biosciences (Shanghai, China). The ionizable lipid C2 was synthesized by the State Key Laboratory of Biotherapy, West China Hospital, Sichuan University.

### Cell culture

DC2.4 and EBV-CT26 were cultured in DMEM medium containing 10% fetal bovine serum, 1% penicillin, and 1% streptomycin, incubated with 5% CO_2_ at 37℃. The medium was changed every two days.

### Animals

Female Balb/c (6–8 weeks old) were purchased from Chongqing Tenxin Bio-Technology Co., Ltd. Mice were fed in a pathogen-free laboratory (SPF) with water and food. The mice were marked uniquely by earmarks in advance. A tumor challenge was performed by injecting 1 × 10^6^ EBV-CT26 cells subcutaneously into each Balb/c mouse. All animal experiments in this study were approved by the Animal Ethics Committee of West China Hospital of Sichuan University.

### Synthesis of the ionizable lipid (C2)

The ionizable lipid C2 used in this study was designed based on four tertiary amino nitrogen atoms (4N4T). The 4N core was synthesized via a Michael addition reaction and deprotection of Boc groups and the four tails were added through a ring-opening reaction of the epoxide. The synthesis route has been described in our previous study [22].

### Preparation of C2@mRNA-LNP

C2, cholesterol, DSPC, and DMG-PEG2000 were dissolved in anhydrous ethanol with a molar ratio of 35/46.5/16/2.5 to prepare an ionizable lipid composite solution with a C2 concentration of 15mg/ml. The mRNA was diluted in a 10 mmol/L citric acid buffer solution composed of citric acid and sodium citrate solution dissolved in enzyme-free water with pH = 6. The ionizable lipid stock solution and the mRNA aqueous solution were mixed using a microfluidic chip with a volume ratio of 1/3 and the mass ratio of C2 /mRNA was 15/1. The mixing speed was 12 ml/min. The concentration of mRNA was 0.25 mg/ml, which was diluted with citrate buffer solution to the concentration of administration (0.1 mg/ml). The microstructure of LNP was observed under transmission electron microscopy (TEM).

### Characterization of LNP

The particle size and potential of LNP were measured by Malvern Laser Particle Size Analyzer (Zetasizer Nano ZS 90, Malvern, UK)) at 25 °C.

### Encapsulation efficiency of C2@mLMP2

The encapsulation efficiency of the sample was analyzed by Stunner high-throughput concentration particle size analyzer.

### Transfection efficiency of C2@eGFP mRNA

DC2.4 cells were cultured and transferred into six-well plates one day in advance. On the second day, C2@eGFP mRNA was prepared and diluted with citric acid buffer to a concentration of 0.1 mg/ml; the six-well plate was divided into three groups. The first group was used as the control group. The second group was transfected with mRNA (0.5 μg) with Lipo2K reagent. The third group was added with C2@eGFP mRNA (0.5μg). After incubation overnight and being photographed under a fluorescence microscope the next day, the DC cells in the six-well plate were collected with a flow tube to observe the luminescence of the cells in the FITC channel.

### Lysosomal escape ability of C2@Cy5-mRNA

DC2.4 cells in the logarithmic growth phase were spread in confocal dishes (10^5^ cells per dish) with 200 μl medium containing antibiotics and serum, incubated with 5% CO_2_ at 37 °C overnight. Discard the medium, mix 50 μl C2@Cy5-mRNA (0.1 mg/ml) and 150 μl complete medium to the confocal dishes, (50 μl PBS and 150 μl complete medium mixture to the control group), and incubate for 3h. Then LysoTrackerGreen was added to the confocal dishes, incubating for 2h. Cells in the confocal dishes were washed with PBS and fixed for 20 min. Intracellular Staining Permeablization Wash Buffer containing DAPI dye was added, and cells were observed under the confocal microscope after PBS washing.

### Antigen expression in vivo (lymph node targeting effect)

C2@Luc mRNA was prepared according to the method. The mRNA concentration was diluted to 0.2 mg/ml with citric acid buffer and inoculated subcutaneously in mice. Each animal was given 30 μg of mRNA. After 6 h of administration, each animal was intraperitoneally injected with 200 μl PBS-dissolved solution containing 3 mg of luciferase substrate. Ten minutes later, the animals were placed supine under anesthesia and observed using live imaging.

### Flow cytometry

Tumor tissues of mice in each group were collected to prepare single-cell suspension at a density of 10^6^ cells/wells (5 samples per group). Antibodies targeting CD45, CD3, CD8, CD279, and TIGIT were used to label exhausted CD8 + T cells with 1 μl per sample. Spleen tissues of mice in each group were collected to prepare single-cell suspension (5 samples per group). Antibodies targeting CD45, CD3, CD8, CD44, and CD62L were used to label memory CD8 + T cells with 1 μl per sample. Cells were detected using flow cytometry.

### Pathological examination

The heart, liver, spleen, lung, kidney, and tumor tissues were fixed using formaldehyde and embedded in paraffin. H&E staining of the tissue slices was performed for pathological analysis.

### Statistical analysis

Tumor volume was calculated as V = length×width^2^/2. Differences in tumor growth and body weight curve among the four groups were tested using Two Way ANOVA and Tukey’s multiple comparisons tests. Cell numbers in flow cytometry were compared using the Mann–Whitney test. All the analyses.

## Results and discussions

### Characterization of C2@mLMP2

**Fig. 1:**
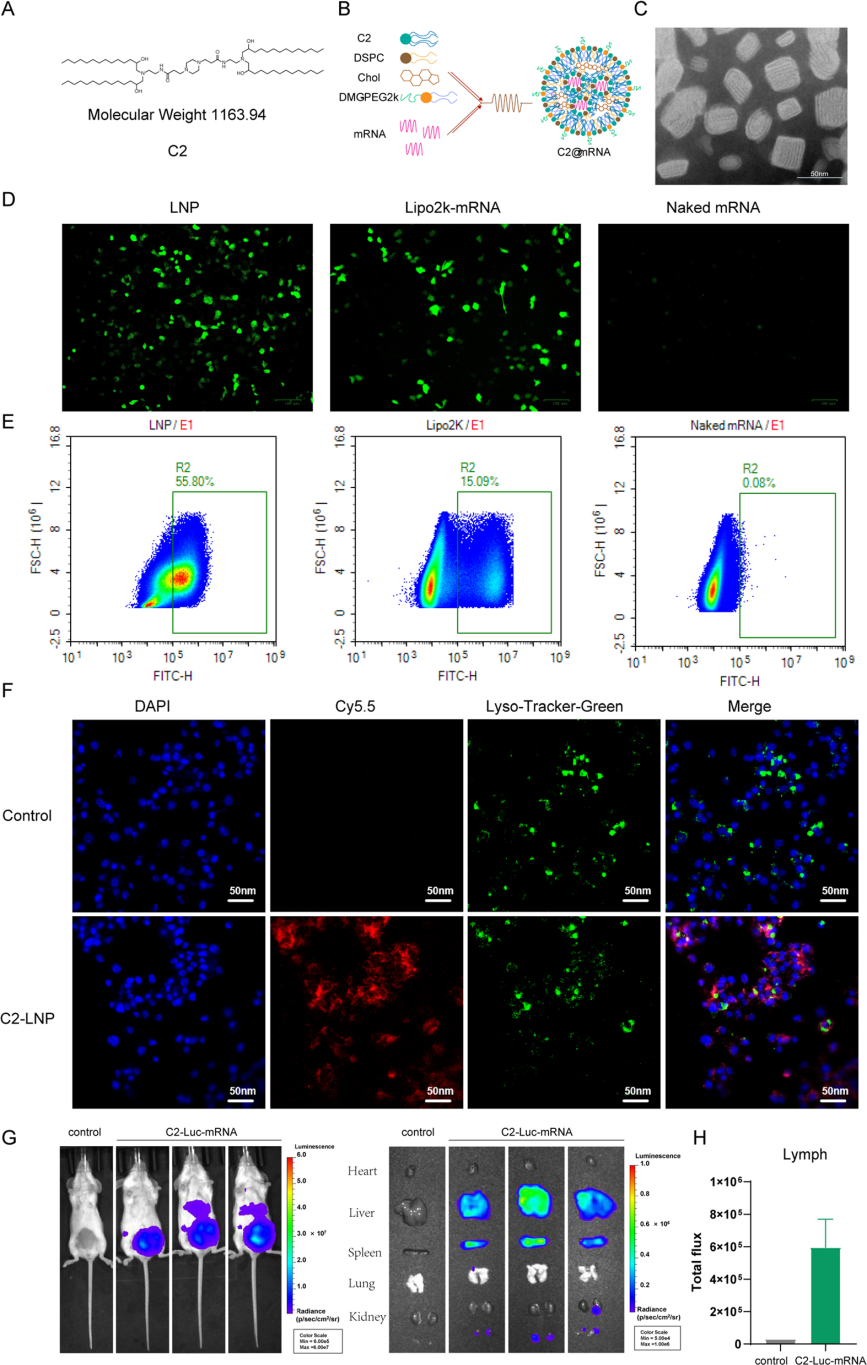
Characterization of C2@mLMP2. **A** Chemical structure of C2. **B** Preparation of C2@mRNA-LNP. **C** TEM image of C2@mLMP2. **D** Fluorescence microscope of DC2.4 cells transfected by C2@mRNA, Lipo2k@mRNA, and naked mRNA. **E** Luminescence of the transfected DC2.4 cells in the FITC channel. **F** Cy5 mRNA in C2@Cy5-mRNA escapes from lysosomes sufficiently after administration in DC2.4 cells. **G**, **H** Antigen expression in vivo

The preparation of C2@mLMP2 was displayed in Materials and Methods (Fig. 1A, B). Figure 1C showed that C2@mRNA exhibited a multilayer capsule structure, indicating the formation of lipid nanoparticles, which is also an important feature of nanoliposomes. The particle size of the nanomaterial was measured to be 97.60 nm, and the zeta potential was-2 mV (Additional file 1: Fig. S1). The encapsulation efficiency was 95.6% by Stunner high-throughput particle measurement analysis. The effect of C2 on mRNA expression at the cellular level was detected in DC2.4 cells. The expression ability of C2 nanoliposomes on mRNA delivery was stronger compared with Lipo2K (Fig. 1D), and the flow cytometry showed a higher proportion of cells with fluorescent expression in the C2@eGFP-mRNA group (Fig. 1E), which indicates an enhanced intracellular expression of mRNA synthesized in vitro. Then we tested the lysosomal escape ability of C2@mRNA. Figure 1F shows that Cy5 mRNA in C2@Cy5-mRNA can escape from lysosomes sufficiently after administration in DC2.4 cells, which indicates that during the delivery of mRNA, C2 nanoliposomes can release mRNA from lysosomes to be translated in the cytoplasm. The in vivo distribution assay was performed using live imaging. The results showed that the fluorescence in the administered mice was mainly distributed in the liver, spleen, and lymph nodes (bilateral abdominal lymph nodes) (Fig. 1G, H). The successful delivery of mRNA to tumor-draining lymph nodes enables the activation of antitumor immunity through antigen-presenting cells, and the vaccine mainly performs immune response through liver metabolism and spleen.

### The combination of C2@mLMP2 and αPD-1 provoked a strong anti-tumor effect

**Fig. 2:**
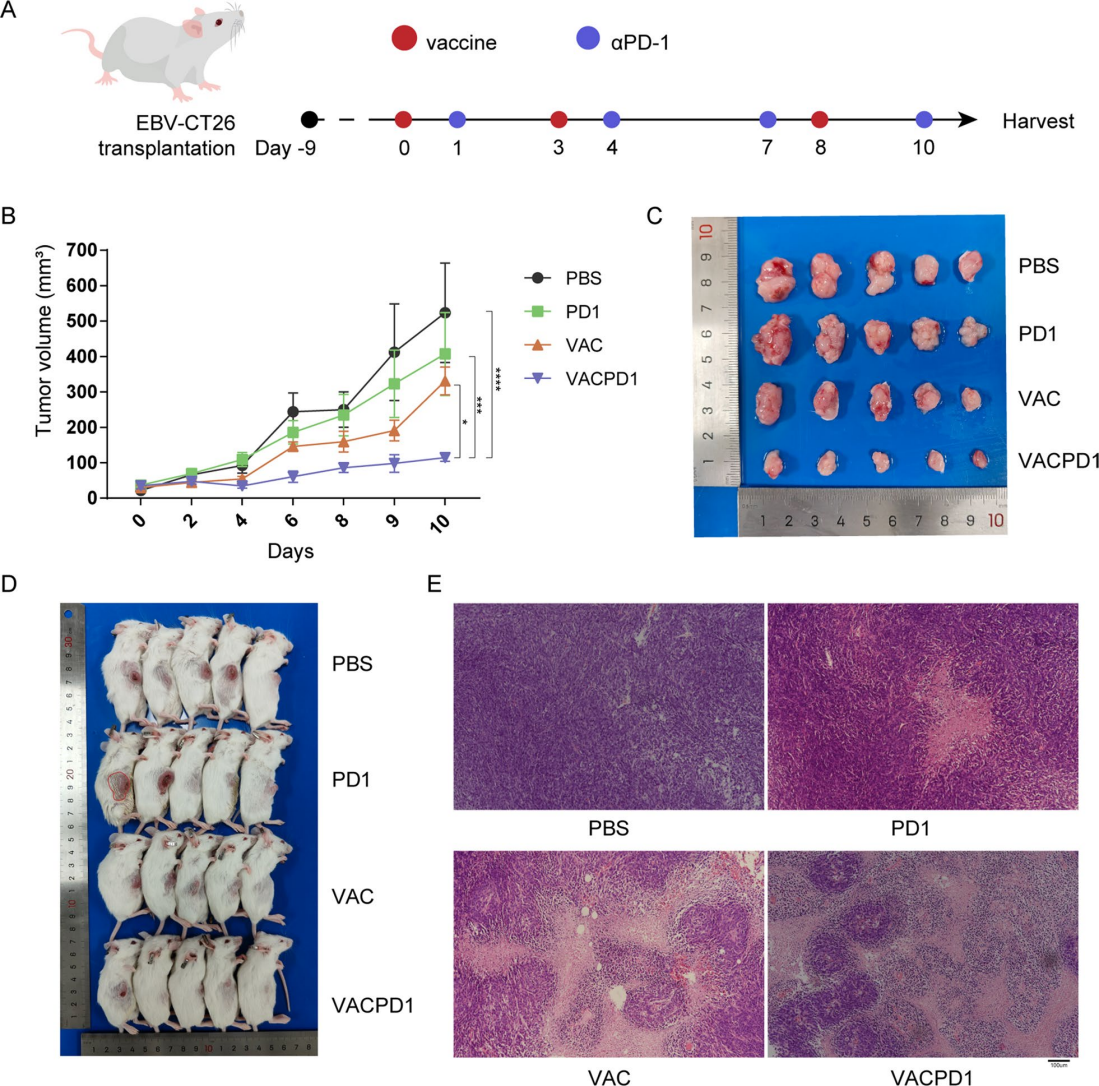
Anti-tumor effect of C2@mLMP2 combing αPD-1. **A** Experimental flow. **B** The Tumor growth curve of the four groups (Tukey’s multiple comparisons test p-value: **** < 0.0001 < *** < 0.001 < ** < 0.01 < * < 0.05). **C** Tumors images on Day 11. **D** Mice images on Day 11. **E** H&E staining of tumor tissues

EBV proteins EBNA1, LMP1, and LMP2 are expressed in most EBV + NPC tumors and play a key role in the transformation of normal cells into cancer cells [23, 24]. Targeting EBNA1, LMP1 or LMP2 has become an effective way in the treatment of NPC through vaccines. To test the in vivo effect of C2@mLMP2 and its synergy effect with αPD-1, we constructed a cell line (EBV-CT26) expressing EBNA1, LMP1, and LMP2. CT26 was used as a template because it shows a poor response to αPD-1 treatment. When the tumors were measurable with an average volume of 30–50 mm^3^ (Day 0), the mice were randomly divided into four treatment groups: 1. Control group (PBS injection); 2. VAC group (Subcutaneous injection of C2@mLMP2 at Day 0, 3, 8 with 15ug mLMP2); 3. PD1 group (Intraperitoneal injection of 100μg αPD-1 at Day 1, 4, 7, 10); 4. VAC + PD1 group (Combined treatment of VAC group and PD1 group) (Fig. 2A). Tumor volume was recorded every two days since Day 0. As shown in Fig. 2B, single vaccination achieved a better tumor control compared with the PBS group, although with a P value of 0.0517 using Tukey’s multiple comparisons test after two-way ANOVA. While the combination of C2@mLMP2 and αPD-1 significantly enhanced the anti-tumor effect, compared with the control group or each single-treatment group (Fig. 2B–D). H&E staining of tumor tissues verified a pronounced tumor cell apoptosis in the VAC + PD1 group (Fig. 2E).

Although the high PD-L1 expression rate and TIL infiltration TME make NPC suitable for αPD-1 therapy, it faces other challenges like high tumor heterogeneity, high recurrence rate, and complicated immunosuppressive factors including neoantigen losing, MHC aberration, DC incapacity, Tregs infiltration and T cell exhaustion caused by multiple checkpoints [15]. Clinical use of αPD-1 in NPC is still limited in local recurrence and/or metastatic NPC, while monotherapy of αPD-1 is mainly used as a second- or late-line strategy after platinum-based chemotherapy. The combination of αPD-1 and chemotherapy achieves a synergy effect since platinum and 5-FU can promote the presentation of neoantigen and alleviate the immunosuppressive environment [25–27]. Radiation therapy, which is the main treatment for NPC, can also enhance tumor response to αPD-1 through immunogenic cell death that releases neoantigen but it also leads to upregulation of Tregs and expression of immune checkpoints. Since EBV plays an important role in NPC, targeting EBV protein using vaccines may be promising way to enhance the αPD-1 effect. The C2@mLMP2 vaccine was designed to target tumor-draining lymph nodes to achieve a strong immune activation with limited systemic side effects. The result indicated that C2@mLMP2 can apparently improve the tumor response to αPD-1.

### C2@mLMP2 enhanced the anti-tumor effect of αPD-1 by reversing CD8 + T cell exhaustion

**Fig. 3:**
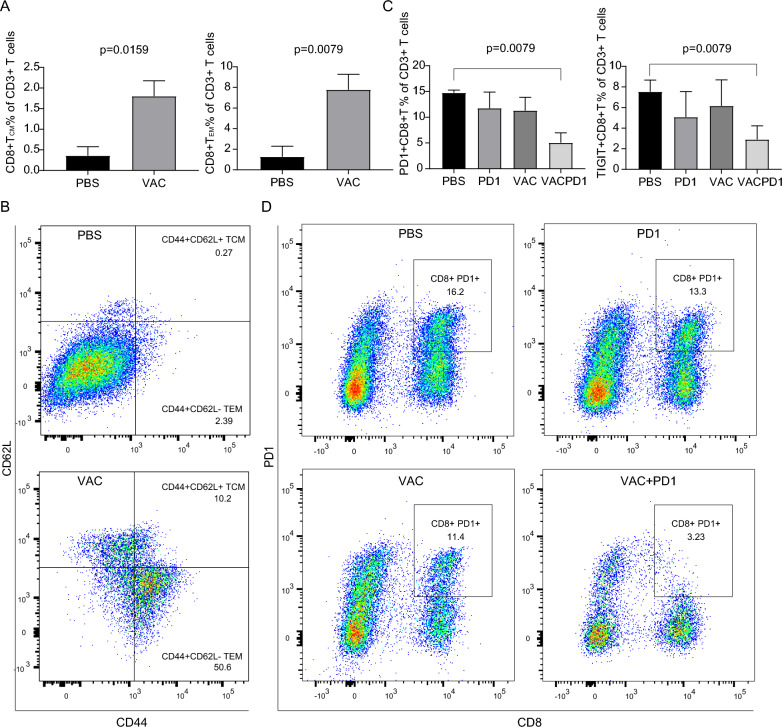
Flow cytometry results of the in vivo experiment. **A** CD8 + TCM% (CD8 + TEM%) of CD3 + T cells in spleens from the PBS and VAC group. **B** Typical flow cytometry graphs of **A**. **C** PD1 + CD8 + T % (TIGIT + CD8 + T %) of CD3 + T cells in tumors from the PBS, PD1, VAC, and VACPD1 groups. **D**, Typical flow cytometry graphs of **C**

We performed flow cytometry to examine the phenotypic alterations of immune cells in tumors and spleens from different groups. The memory T cell ratios in the spleen between mice receiving C2@mLMP2 and PBS were compared. As shown in Fig. 3A, B, both CD8 + central memory T (Tcm) cells and CD8 + effective memory T (Tcm) cells of the CD3 + T cells ratios in the spleen were significantly increased in mice receiving C2@mLMP2 treatment. The immunological memory effect is one of the advantages of tumor vaccines, especially for tumors with a high recurrence rate like NPC. The memory effect of Tcm can persist for years. Compared with naïve T cells, the activation threshold of memory T cells is much lower and the response time is shorter. Memory T cells also show higher migration ability toward lymph nodes owing to high expression of CCR7, a lymph node homing receptor [28]. To understand the mechanism of the synergistic effect, we characterized tumor resident CD8 + T cells of the four groups. The result showed that the exhausted CD8 + T cells ratio in CD3 + T cells marked by PD-1 and TIGIT was significantly decreased in mice from the combination therapy group (Fig. 3C, D). T-cell exhaustion is commonly caused by the persistence of antigens and inflammation [29]. Compared with memory T cells and effective T cells, exhausted T cells lose effector function and exhibit enhanced and sustained expression of multiple immune checkpoints [29, 30]. αPD-1 is designed to block the immune checkpoint of PD-1 and its ligand PD-L1/2 to restore the function of CD8 + T cells. However, reversing T cell exhaustion using αPD-1 only works when part of the T cells are not completely terminal [29]. Monotherapy may not be an effective strategy considering the complexity of the exhaustion mechanism. In this study, we observed a strong synergy effect of exhaustion reversing when we combined αPD-1 with a lymph node-directed EBV-mRNA vaccine.

### Safety of C2@mLMP2 combining αPD-1 in vivo

**Fig. 4:**
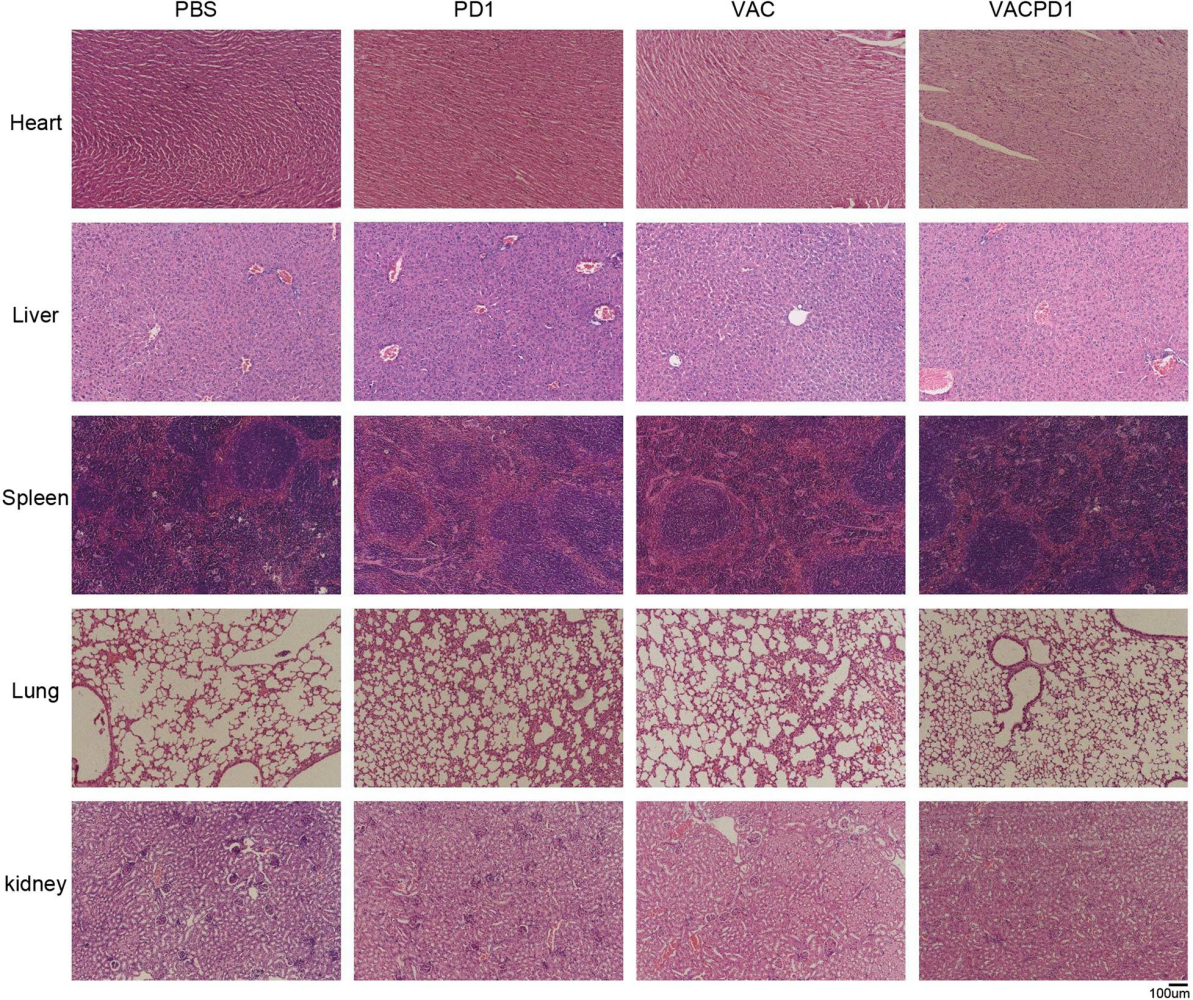
Safety of C2@mLMP2 combining αPD-1 in vivo. H&E staining of the heart, liver, spleen, lung, and kidney of mice from different groups

To evaluate the safety of C2@mLMP2 and its combination with αPD-1, we monitored the body weight of mice during the treatment. As shown in the Additional file 1, the body weights of mice from each group did not show a difference from Day 0 to tumor harvest (Additional file 1: Fig. S2). We further collected the heart, liver, spleen, lung, and kidney to perform H&E staining. No significant histopathological changes like necrosis, inflammation, or structural destruction were observed in organs of the VAC and VACPD1 groups (Fig. 4). The result indicates that C2@mLMP2 combing with αPD-1 is safe in vivo. Compared with other forms of vaccines, mRNA-based vaccines are well tolerated with adverse events being manageable and transient [19]. The mRNA is delivered to the cytoplasm without integration into the host genome, and it is easily degraded which also reduces the toxicity. LNP makes mRNA vaccines more selective by preventing non-specific uptake in healthy tissues.

## Conclusions

This is the first study proving the synergistic effect of the EBV-mRNA vaccine and PD-1 inhibitors for EBV-related tumors. We developed an LMP2-mRNA vaccine based on ionizable lipid nanoparticles to deliver the vaccine to lymph nodes. The strong synergistic effect with αPD-1 and its safety was verified by animal experiments. This study provides theoretical evidence for further clinical trials that may expand the application scenario and efficacy of immunotherapy in EBV-related tumors like NPC.

### Supplementary Information

**Additional file 1.** The particle size and the zeta potential of the nanomaterial.

**Additional file 2.** Body weight of the mice in the four groups.

## Data Availability

The data generated during the current study are available from the corresponding author on reasonable request.

## References

[1] Yang T (2022). EBV infection and its regulated metabolic reprogramming in nasopharyngeal tumorigenesis. Front Cell Infect Microbiol.

[2] Bu G-L, Xie C, Kang Y-F, Zeng M-S, Sun C (2022). How EBV infects: the tropism and underlying molecular mechanism for viral infection. Viruses.

[3] Yin H, Qu J, Peng Q, Gan R (2019). Molecular mechanisms of EBV-driven cell cycle progression and oncogenesis. Med Microbiol Immunol.

[4] Xiao Z, Chen Z (2019). Deciphering nasopharyngeal carcinoma pathogenesis via proteomics. Expert Rev Proteomics.

[5] Zhu Q (2021). Advances in pathogenesis and precision medicine for nasopharyngeal carcinoma. MedComm.

[6] Kang Y (2020). Advances in targeted therapy mainly based on signal pathways for nasopharyngeal carcinoma. Signal Transduct Target Ther.

[7] Bossi P (2021). Nasopharyngeal carcinoma: ESMO-EURACAN clinical practice guidelines for diagnosis, treatment and follow-up†. Ann Oncol.

[8] Yarza R, Bover M, Agulló-Ortuño MT, Iglesias-Docampo LC (2021). Current approach and novel perspectives in nasopharyngeal carcinoma: the role of targeting proteasome dysregulation as a molecular landmark in nasopharyngeal cancer. J Exp Clin Cancer Res.

[9] Ge Y (2020). In vitro evaluation of the therapeutic effectiveness of EBV-LMP2 recombinant adenovirus vaccine in nasopharyngeal carcinoma. Biomed Pharmacother.

[10] Li W (2022). Immunotherapeutic approaches in EBV-associated nasopharyngeal carcinoma. Front Immunol.

[11] Lee HM, Okuda KS, González FE, Patel V (2019). Current perspectives on nasopharyngeal carcinoma. Adv Exp Med Biol.

[12] Ding R-B (2021). Molecular landscape and subtype-specific therapeutic response of nasopharyngeal carcinoma revealed by integrative pharmacogenomics. Nat Commun.

[13] Guan S, Wei J, Huang L, Wu L (2020). Chemotherapy and chemo-resistance in nasopharyngeal carcinoma. Eur J Med Chem.

[14] Liu J, Zeng Z, Wang D, Qin G (2022). Minimally invasive surgery for early-stage nasopharyngeal carcinoma. J Craniofac Surg.

[15] Xu J-Y, Wei X-L, Wang Y-Q, Wang F-H (2022). Current status and advances of immunotherapy in nasopharyngeal carcinoma. Ther Adv Med Oncol.

[16] Bian J, Niu Y, Ma Y, Chen F, Ma N (2022). A review on the application of PD-1 blockade in EBV-associated nasopharyngeal carcinoma immunotherapy. Appl Bionics Biomech.

[17] Wang F-H (2021). Efficacy, safety, and correlative biomarkers of toripalimab in previously treated recurrent or metastatic nasopharyngeal carcinoma: a phase II clinical trial (POLARIS-02). J Clin Oncol.

[18] Sato H (2020). Investigation of the efficacy and safety of nivolumab in recurrent and metastatic nasopharyngeal carcinoma. In Vivo.

[19] Lorentzen CL, Haanen JB, Met Ö, Svane IM (2022). Clinical advances and ongoing trials of mRNA vaccines for cancer treatment. Lancet Oncol.

[20] Cheng Z, Que H, Chen L, Sun Q, Wei X (2022). Nanomaterial-based drug delivery system targeting lymph nodes. Pharmaceutics.

[21] Chen J (2022). Lipid nanoparticle-mediated lymph node–targeting delivery of mRNA cancer vaccine elicits robust CD8+ T cell response. Proc Natl Acad Sci U S A.

[22] Chen K (2022). mRNA vaccines against SARS-CoV-2 variants delivered by lipid nanoparticles based on novel ionizable lipids. Adv Funct Mater.

[23] Münz C (2000). Human Cd4+ T lymphocytes consistently respond to the latent epstein-barr virus nuclear antigen EBNA1. J Exp Med.

[24] Brooks L, Yao QY, Rickinson AB, Young LS (1992). Epstein-Barr virus latent gene transcription in nasopharyngeal carcinoma cells: coexpression of EBNA1, LMP1, and LMP2 transcripts. J Virol.

[25] Salewski I (2021). Combined gemcitabine and immune-checkpoint inhibition conquers anti-PD-L1 resistance in low-immunogenic mismatch repair-deficient tumors. Int J Mol Sci.

[26] Chen Y-L, Chang M-C, Cheng W-F (2017). Metronomic chemotherapy and immunotherapy in cancer treatment. Cancer Lett.

[27] McDonnell AM (2015). Tumor-infiltrating dendritic cells exhibit defective cross-presentation of tumor antigens, but is reversed by chemotherapy. Eur J Immunol.

[28] Liu Q, Sun Z, Chen L (2020). Memory T cells: strategies for optimizing tumor immunotherapy. Protein Cell.

[29] Wherry EJ (2011). T cell exhaustion. Nat Immunol.

[30] Keir ME, Butte MJ, Freeman GJ, Sharpe AH (2008). PD-1 and its ligands in tolerance and immunity. Annu Rev Immunol.

