# Supplementary figures and images for: LMP2-mRNA lipid nanoparticle sensitizes EBV-related tumors to anti-PD-1 therapy by reversing T cell exhaustion
Source: J Nanobiotechnology. 2023 Sep 8;21:324. doi: 10.1186/s12951-023-02069-w (PMC10486025; doi:10.1186/s12951-023-02069-w)

Size Distribution by Intensity

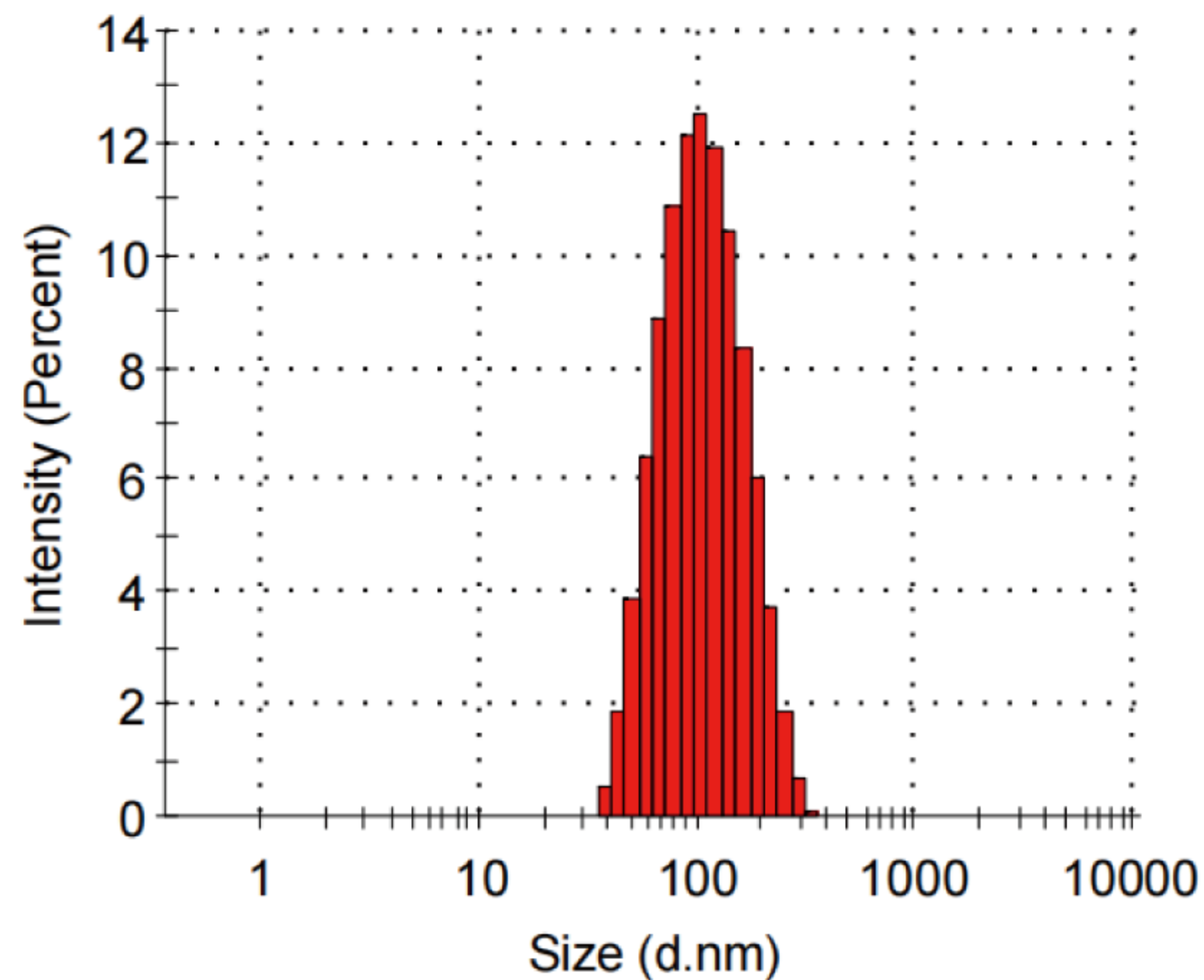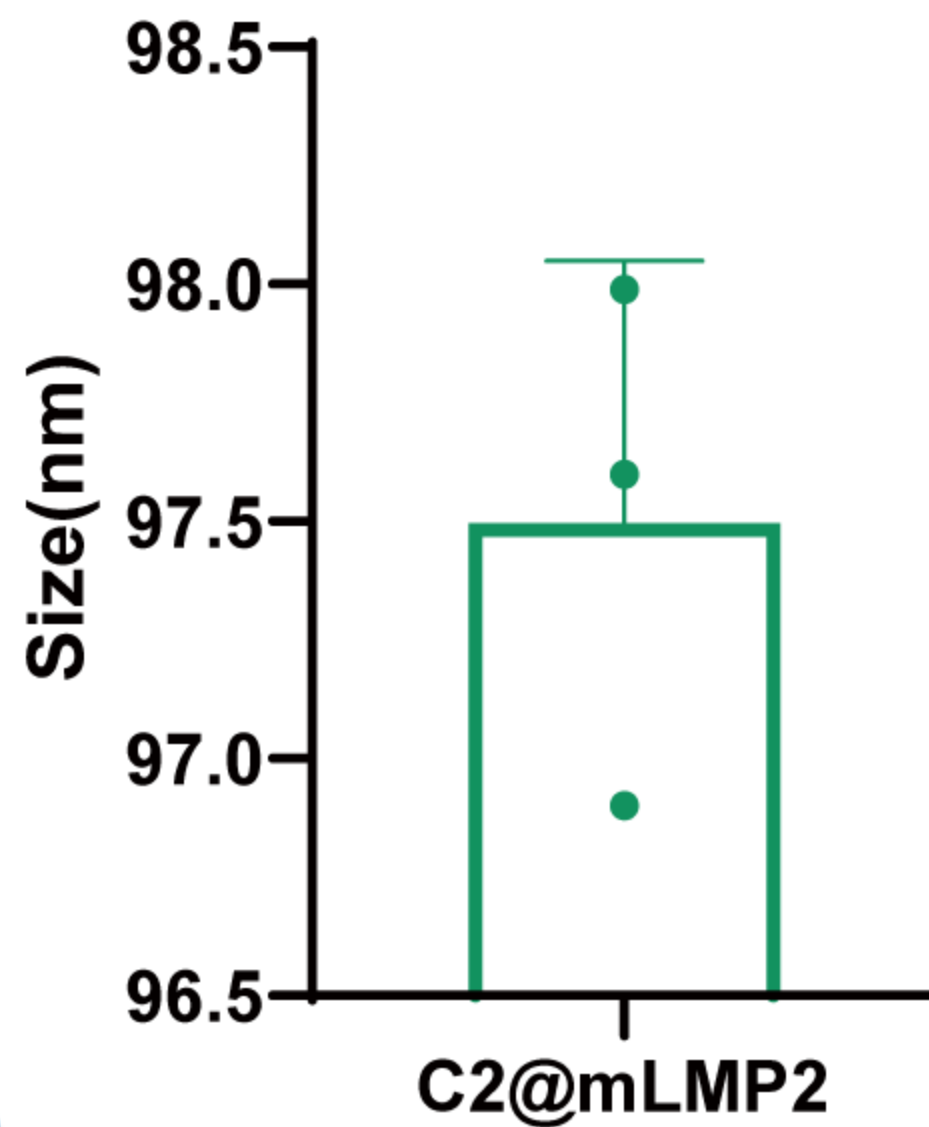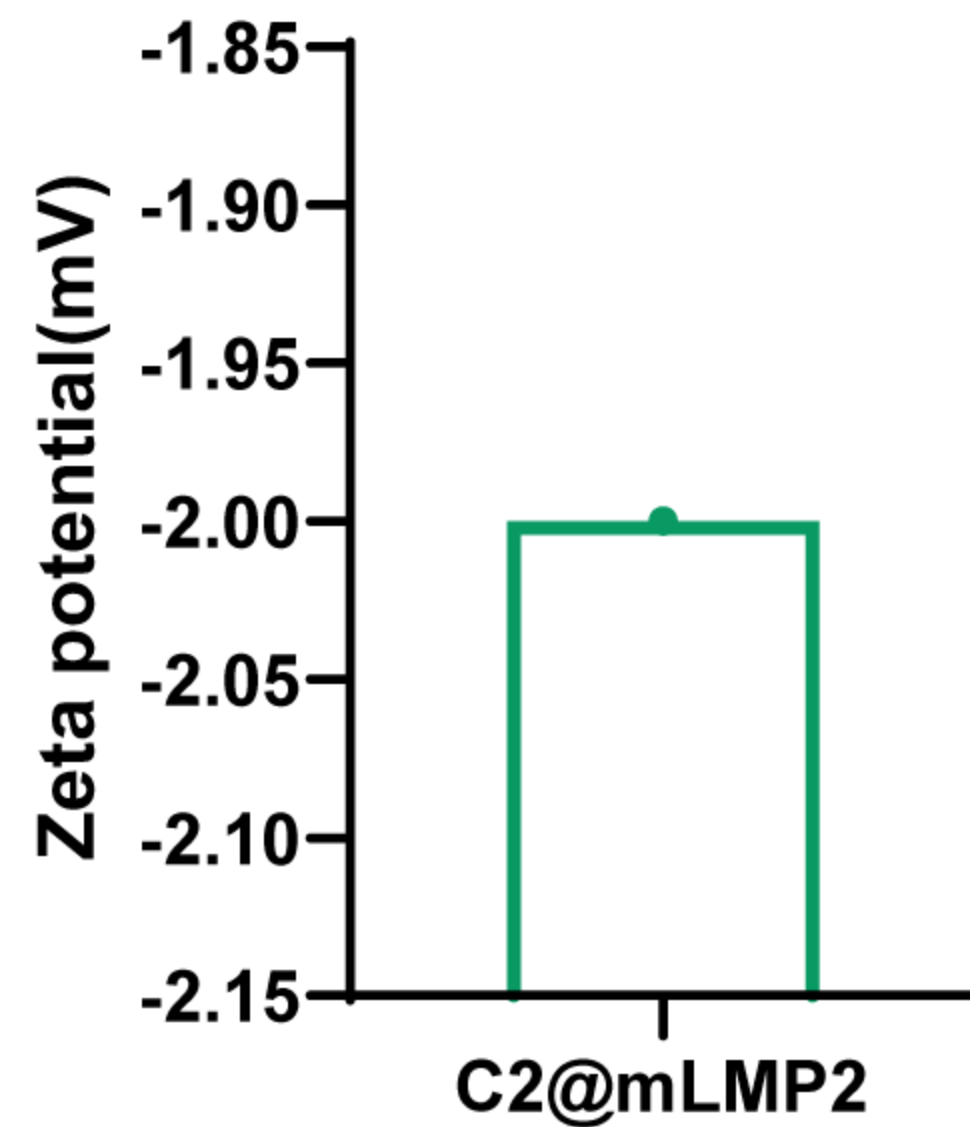

sufficient characterization of the nanoparticles

Supplement: Supplementary file 1 — Additional file 1. The particle size and the zeta potential of the nanomaterial. [file 12951_2023_2069_MOESM1_ESM.pdf]

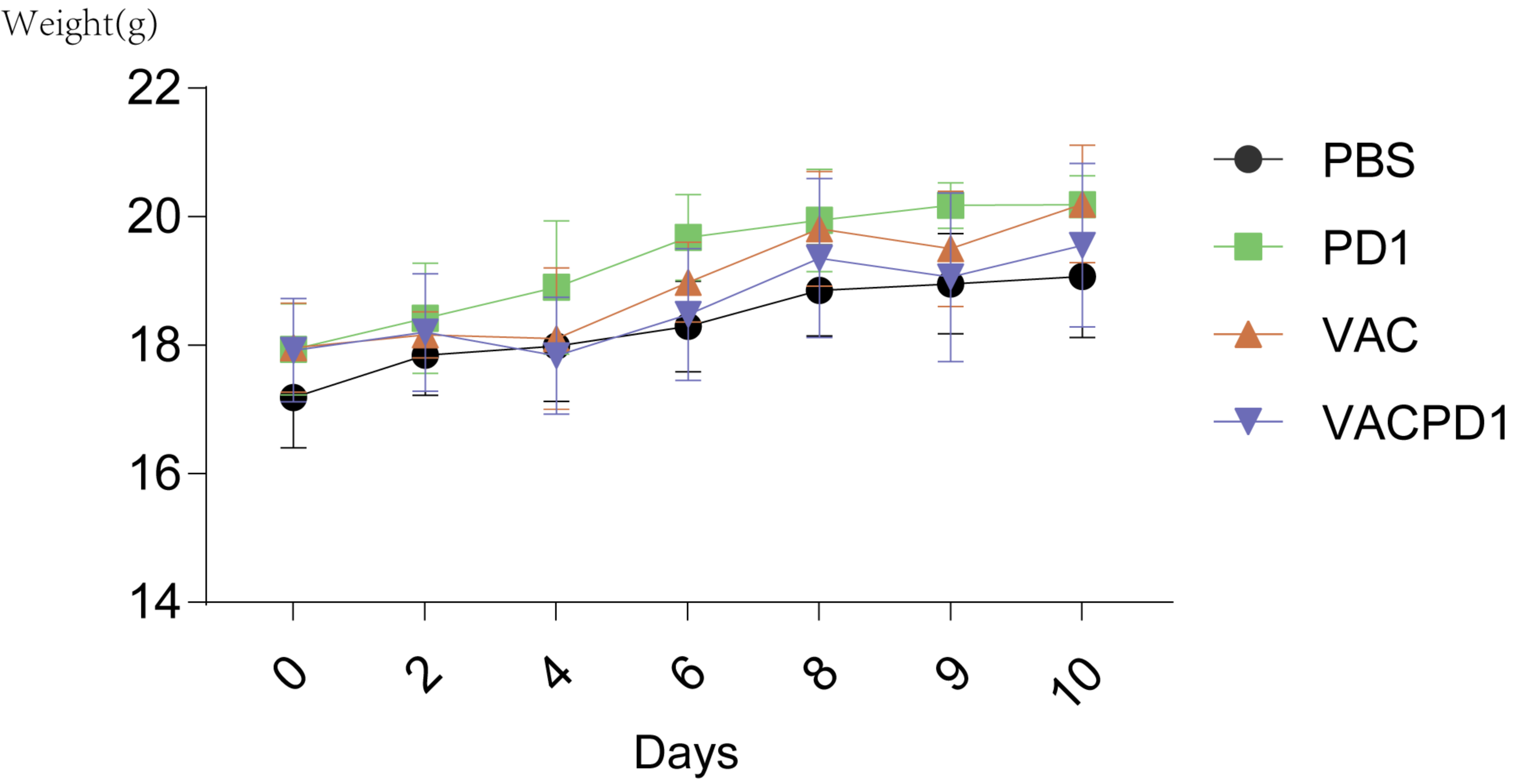

Supplement: Supplementary file 2 — Additional file 2. Body weight of the mice in the four groups. [file 12951_2023_2069_MOESM2_ESM.pdf]
